# Perfluorinated Compounds, Polychlorinated Biphenyls, and Organochlorine Pesticide Contamination in Composite Food Samples from Dallas, Texas, USA

**DOI:** 10.1289/ehp.0901347

**Published:** 2010-02-10

**Authors:** Arnold Schecter, Justin Colacino, Darrah Haffner, Keyur Patel, Matthias Opel, Olaf Päpke, Linda Birnbaum

**Affiliations:** 1 Division of Environmental and Occupational Health Sciences, University of Texas School of Public Health, Dallas, Texas, USA; 2 Department of Environmental Health Sciences, University of Michigan School of Public Health, Ann Arbor, Michigan, USA; 3 University of Texas Southwestern Medical School, Dallas, Texas, USA; 4 Eurofins GfA GmbH, Hamburg, Germany; 5 National Institute of Environmental Health Sciences, National Institutes of Health, Department of Health and Human Services, Research Triangle Park, North Carolina, USA

**Keywords:** food, PCBs, pesticides, PFCs, United States

## Abstract

**Objectives:**

The objective of this article is to extend our previous studies of persistent organic pollutant (POP) contamination of U.S. food by measuring perfluorinated compounds (PFCs), organochlorine pesticides, and polychlorinated biphenyls (PCBs) in composite food samples. This study is part of a larger study reported in two articles, the other of which reports levels of polybrominated diphenyl ethers and hexabromocyclododecane brominated flame retardants in these composite foods [Schecter et al. 2010. Polybrominated diphenyl ethers (PBDEs) and hexabromocyclodecane (HBCD) in composite U.S. food samples, Environ Health Perspect 118:357–362].

**Methods:**

In this study we measured concentrations of 32 organochlorine pesticides, 7 PCBs, and 11 PFCs in composite samples of 31 different types of food (310 individual food samples) purchased from supermarkets in Dallas, Texas (USA), in 2009. Dietary intake of these chemicals was calculated for an average American.

**Results:**

Contamination varied greatly among chemical and food types. The highest level of pesticide contamination was from the dichlorodiphenyltrichloroethane (DDT) metabolite *p*,*p′-* dichlorodiphenyldichloroethylene, which ranged from 0.028 ng/g wet weight (ww) in whole milk yogurt to 2.3 ng/g ww in catfish fillets. We found PCB congeners (28, 52, 101, 118, 138, 153, and 180) primarily in fish, with highest levels in salmon (PCB-153, 1.2 ng/g ww; PCB-138, 0.93 ng/g ww). For PFCs, we detected perfluorooctanoic acid (PFOA) in 17 of 31 samples, ranging from 0.07 ng/g in potatoes to 1.80 ng/g in olive oil. In terms of dietary intake, DDT and DDT metabolites, endosulfans, aldrin, PCBs, and PFOA were consumed at the highest levels.

**Conclusion:**

Despite product bans, we found POPs in U.S. food, and mixtures of these chemicals are consumed by the American public at varying levels. This suggests the need to expand testing of food for chemical contaminants.

Persistent organic pollutants (POPs) are chemicals that are resistant to degradation in the environment and biota, bioaccumulate, and are toxic. Perfluorinated compounds (PFCs) have been widely used in consumer applications for the past 50 years. Because of their excellent surfactant properties, they are used as stain, grease, or water repellants. The most widely used PFC, perfluorooctanesulfonic acid (PFOS), has been phased out by its major U.S. manufacturer, 3M (Lau et al. 2007). Despite this, PFOS is widely detected in the environment, animals, and humans. Toxicologic studies have reported that chronic exposure to PFCs, including PFOS and perfluorooctanoic acid (PFOA), which is not technically a POP because of its short half-life, can lead to hepatotoxicity, including increased liver weight, fluctuations in liver enzyme levels, and hepatocarcinoma (Son et al. 2008). Rodents exposed to PFOS have experienced adverse developmental outcomes, including decreased rates of weight gain and delayed lung maturation (Grasty et al. 2003; Lau et al. 2003). Epidemiologic studies have associated elevated levels of PFCs with decreased fecundity and impaired sperm quality (Fei et al. 2009; Joensen et al. 2009). Dietary intake may be a major route of exposure to PFCs because they have been detected in food from various countries (Ericson et al. 2008). PFCs have also been detected in air, dust, water, and soil (Bjorklund et al. 2009; Dreyer et al. 2009; Post et al. 2009; Renner 2009). PFOA and PFOS have been detected in > 98% of people tested in a representative sample of the U.S. population (Calafat et al. 2007).

Organochlorine pesticides such as dichlorodiphenyltrichloroethane (DDT) were widely used before recognition of their toxicity and persistence. Pesticide exposure has been associated with arthritis, breast cancer, and diabetes (Cox et al. 2007; Dorgan et al. 1999; Lee et al. 2007). Organochlorine exposure has been associated with neurobehavioral changes and DNA hypomethylation (Jurewicz and Hanke 2008; Rusiecki et al. 2008). The main nonoccupational route of exposure to organochlorines is through dietary intake (Brock et al. 1998).

Polychlorinated biphenyls (PCBs) were used in electrical systems and in hydraulic fluid. Their production was banned in the United States in the 1970s (Garmon 1982). However, PCBs are still detectable in wildlife and humans (Brown et al. 2006; Meeker et al. 2007). PCBs have been associated with adverse neurologic development, including decreases in motor skills and cognitive development (Ribas-Fito et al. 2001). Prenatal exposure to PCBs and related chemicals, the chlorinated dibenzofurans, in highly exposed populations has been associated with altered pubertal timing and growth abnormalities, including decreased height and birth weight (Guo et al. 1995). Cancer and endocrine disruption have been associated with adult exposure (Agency for Toxic Substances and Disease Registry 2000).

In this study, we expanded our previous market basket surveys that measured levels of POPs in U.S. foods (Schecter et al. 1994, 2004, 2006, 2009) to include organochlorine pesticides, PCBs, and PFCs and explored dietary intake as a route of exposure for the general U.S. population to these chemicals. This study is part of a larger study reported in two articles, the other of which reports levels of polybrominated diphenyl ethers (PBDEs) and hexabromocyclododecane (HBCD) brominated flame retardants in these composite foods (Schecter et al. 2010).

## Materials and Methods

### Sample collection

Ten individual samples of 31 commonly consumed food types (310 samples total) were collected from five Dallas, Texas (USA), grocery stores in 2009. Foods purchased were chosen to match those measured in our previous market basket surveys, with the addition of some other commonly consumed foods (Schecter et al. 2004, 2006). Samples were meat products (ground beef, bacon, turkey, sausage, ham, chicken breast, roast beef, canned chili), fish (salmon, canned tuna, catfish, tilapia, cod, canned sardines in water, frozen fish sticks), dairy (butter, milk, cream cheese, American cheese, other cheeses—mozzarella, Colby, cheddar, Swiss, provolone, and Monterey jack—ice cream, frozen yogurt, yogurt), vegetable-based foods (olive oil, canola oil, margarine, cereal, apples, potatoes, peanut butter), and eggs. Perishable samples were frozen at −80°C before shipping on dry ice to Eurofins GfA GmbH (Hamburg, Germany) for chemical analysis. Nonperishable samples were stored and shipped at room temperature. Equal weights of each of the 10 samples for each food type were combined to form a composite sample to estimate average levels of chemical contamination in common U.S. foods.

### Chemical analysis

#### Perfluorinated compounds

Analyses were performed using the isotope dilution method. Analytical standards were obtained from Wellington Laboratories (Guelph, ON, Canada): native standards [PFOS, PFOA, perfluorobutane sulfonate (PFBS), perfluorohexansulfonate (PFHxS), perfluorohexanoic acid (PFHxA), perfluoroheptanoic acid (PFHpA), perfluorononanoic acid (PFNA), perfluorodecanoic acid (PFDA), perfluorooctanosulfonamide (PFOSA), perfluorodecane sulfonic acid (PFDeS), and perfluorododecanoic acid (PFDoA)] and three internal ^13^C-labeled standards (^13^C_4_-PFOS, ^13^C_4_-PFOA, and ^13^C_8_-PFOA). All samples were freeze-dried, and a mixture of two ^13^C-labeled PFC congeners (^13^C_4_-PFOS, ^13^C_8_-PFOA) was added to the homogenized fraction of the dried samples before ultrasonic extraction. Extraction in a centrifuge tube (polypropylene) was done twice with methanol. Extract cleanup was performed by solid-phase extraction (Strata-X-AW; Phenomenex, Torrance, CA, USA). The final extract was reduced in volume by a stream of nitrogen; the final volume was 100 μL, containing ^13^C_4_-labeled PFOA used as a recovery standard. The measurements were performed using liquid chromatography/electrospray ionization with tandem mass spectrometry detection using a security guard cartridge (C18 × 2.0 mm inner diameter; Phenomenex) and a Synergy 4U Fusion RP C-18 column (100 mm × 2.0 mm inner diameter, 80A; Phenomenex) for liquid chromatographic separation. Recoveries for the internal standards were between 65% and 105%, with a standard error of 1.8% and 2.5% for ^13^C-PFOA and ^13^C-PFOS, respectively.

Reduction and control of blank data are important steps in quality control when analyzing PFCs at ultratrace levels. Solvents, reagents, materials and equipment were tested before the laboratory procedures and were found to have no PFCs in them. No polytetrafluoroethylene equipment was used. We verified the accuracy of the analytical method by analyzing a certified reference material, and a laboratory blank was run with each batch of 10 samples. Recoveries for validation were between 70% and 110%. The perfluorinated chemicals analyzed were PFOS, PFOA, PFBS, PFHxS, PFHxA, PFHpA, PFNA, PFDA, PFDeS, and PFDoA.

#### Pesticides and PCBs

Analyses were performed using the isotope dilution method. For components analyzed the relevant native standards were used. Wet samples were mixed with sodium sulfate until a free-flowing mixture resulted. Oily samples were treated directly. All samples were spiked with the following ^13^C internal standards: β-hexachlorocyclohexane (β-HCH), γ-HCH, *p*,*p*-DDT, *p*,*p*-dichlorodiphenyldichloroethylene (*p*,*p*-DDE), pentachlorobenzene, hexachlorobenzene, endosulfan sulfate, β-endosulfan, dieldrin, and PCB congeners 28, 52, 101, 118, 138, 153, and 180. Elution of components was performed using a mixture of hexane/acetone.

Cleanup was accomplished by applying a combination of alumina and Florisil columns followed by elution with hexane and toluene. Measurement was by gas chromatography/mass spectrometry (resolution = 10,000) on a 60-m DB5 fused silica column using high-resolution gas chromatography/high-resolution mass spectrometry (Thermo DFS; Thermo Fisher Scientific, Bremen, Germany). As an injection standard, PCB-105 was used. For quantification, a 9-point calibration curve was used. For the toxaphenes, a “sandwich-analysis,” which refers to sample injections between two recalibration solution injections for better precision, was applied. Pesticides analyzed were α-, β-, γ-, δ-, and ɛ-HCH, DDT and DDT metabolites, aldrin, dieldrin, endrin, isodrin, α- and β-endosulfan, endosulfan sulfate, toxaphene (Parlar nos. 26, 50, and 62), heptachlor, mirex, α- and γ-chlordane, oxychlordane, *trans*-nonachlor, *cis*- and *trans*-heptachlor epoxide, and octachlorstyrene. Samples were also analyzed for PCB congeners 28, 52, 101, 118, 138, 153, and 180.

### Dietary intake estimation

We estimated dietary intake from the 2007 U.S. Department of Agriculture (USDA) Food Availability Data Set (USDA 2009). Traditionally, we and others have calculated dietary intake using the 1994–1996 Continuing Survey of Food Intake by Individuals (Huwe and Larsen 2005; Schecter et al. 2004, 2006). However, consumption data during the past 10 years have changed (Adair and Popkin 2005; Nielsen et al. 2002; Popkin and Gordon-Larsen 2004; Popkin et al. 2003). The USDA’s Economic Research Service has published food availability data from 1970 to 2007 (USDA 2009). To account for food spoilage, waste, and losses through transportation and marketing, the USDA also provides loss-adjusted food availability data, to represent daily food consumption for average Americans, in daily grams of food available per person. The USDA data provides per capita daily intake estimations of the various food types measured in this study [see Supplemental Material, Table 1 (doi:10.1289/ehp.0901347)]. To estimate chemical concentrations in beef, we calculated average concentrations of the foods made of or containing beef (ground beef, roast beef, canned beef chili). Similar calculations were done for pork and for fresh and frozen fish. We averaged chemical concentrations in olive oil and canola oil because no separate categories were available in this data set for different types of oil. Concentrations of chemical per food type from this study were multiplied by the loss-adjusted food availability per person for intake estimates. For samples where concentrations of POPs were below the limit of detection (LOD), concentrations were estimated as zero when calculating intake in order to not overestimate intake.

To facilitate intake estimation, pesticides were grouped into HCH compounds (α-HCH, β-HCH, γ-HCH, δ-HCH, ɛ-HCH), DDT and DDT metabolites [*o*,*p*-DDT, *p*,*p′*-DDT, *o*,*p*-DDE, *p*,*p′*-DDE, *o*,*p*-dichlorodiphenyldichloroethane (*o*,*p*-DDD), *p*,*p′*-DDD], endosulfan compounds (α-, β-endosulfan, endosulfan sulfate), toxaphenes (toxaphene-26, toxaphene-50, toxaphene-62), aldrins (aldrin, dieldrin, endrin), heptachlor (heptachlor, *cis*-heptachlor epoxide, *trans*-heptachlor epoxide, *trans*-nonachlor), chlordanes (α-chlordane, β-chlordane, oxychlordane), and chlorobenzenes (pentachlorobenzene, hexachlorobenzene). PCB congeners 28, 52, 101, 118, 138, 153, and 180 were also grouped together for this estimation.

## Results

Tables 1–4 present levels of PFCs, PCBs, and organochlorine pesticides detected. Pesticides detected in < 10% of samples and PCBs and PFCs that we never detected are omitted from the tables (omitted chemicals are PFOS, PFHxA, PFHpA, PFNA, PFDA, PFOSA, PFDeS, PFDoA, γ-HCH, δ-HCH, ɛ-HCH, *o*,*p*-DDT, *o*,*p*-DDE, aldrin, endrin, isodrin, α-endosulfan, β-endosulfan, toxaphene-62, heptachlor, mirex, γ-chlordane, *trans*-heptachlor, octachlorstyrene, and PCB-28).

### PFCs and PCBs

Of the 11 PFCs tested, we detected only PFOA, PFBS, and PFHxS. Concentrations of PFOS and the other PFCs besides PFOA, PFBS, and PFHxS were below the LOD for all 31 foods; LODs ranged from 0.01 ng/g in canned chili to 0.5 ng/g wet weight (ww) in canola oil, with the LOD being the same for each PFC in the individual food types. PFOA contamination was extensive and detected in 17 of 31 food samples (range, 0.02–1.8 ng/g), with no predominance in any food group. In dairy, we detected PFOA only in butter, at a relatively high concentration of 1.07 ng/g ww. Contamination ranged from 0.02 in ham, chicken breast, and canned chili to 1.80 ng/g ww in olive oil. We detected PFBS and PFHxS only in cod (0.12 and 0.07 ng/g ww, respectively).

We detected six of seven tested non-dioxin-like PCB congeners in salmon and canned sardines, with PCB-153 and PCB-138 at highest levels (salmon: PCB-153, 1.2 ng/g ww; PCB-138, 0.93 ng/g ww; canned sardines: PCB-153 and PCB-138, 1.8 ng/g ww each). We measured PCB-153 and PCB-180 in hamburger meat at 1.2 and 0.21 ng/g ww, respectively. PCB-180 was present in peanut butter (0.43 ng/g ww) and ice cream (0.091 ng/g ww).

### Organochlorine pesticides

We found the DDT metabolite *p*,*p′*-DDE most frequently, in 23 of 31 different foods, from 0.041 in whole milk yogurt to 9.0 ng/g ww in fresh catfish fillets. We found high levels in other foods with high fat content: cream cheese (5.7 ng/g ww), butter (5.1 ng/g), American cheese (4.8 ng/g ww), salmon (3.5 ng/g ww), and canned sardines (2.8 ng/g ww).

We detected both dieldrin and *cis*-heptachlor epoxide in 17 and 12 of 31 foods, respectively. Dieldrin concentrations ranged from 0.028 (whole milk yogurt) to 2.3 ng/g ww (fresh catfish fillet). Maximum values of *cis*-heptachlor epoxide were substantially lower than those of dieldrin, from 0.021 ng/g ww (cod) to 0.38 ng/g ww (peanut butter). We did not detect *trans*-heptachlor epoxide in any food.

Salmon was the most contaminated food product, with 24 pesticides detected of the 32 pesticides analyzed; Table 2 lists the 16 pesticides that we detected in > 10% of food samples. For many pesticides detected in salmon, values were relatively high compared with other foods. Compared with meat, dairy, and vegetable products, fish usually was highly contaminated, as was previously reported (Schecter et al. 2004, 2006). Canned sardines were contaminated with 17 of 32, fresh catfish with 16 of 32, and cod with 15 of 32 pesticides tested. Catfish contained relatively high levels of detected pesticides. Peanut butter contained detectable levels of 20 of 32 pesticides.

### Dietary intake

**Figure 1:**
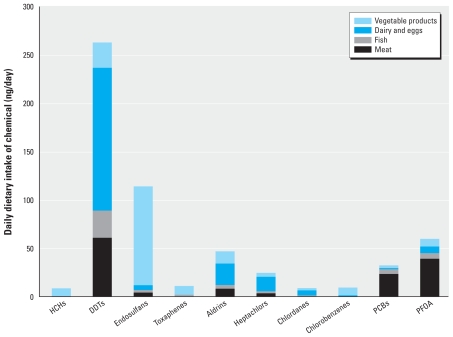
Estimation of per capita dietary exposure to pesticides, PCBs, and PFOA using 2007 USDA food availability data, all ages, estimating values below the LOD as zero. HCHs: α-HCH, β-HCH, δ-HCH, γ-HCH, ɛ-HCH; DDTs: *o*,*p*-DDT, *p*,*p′*-DDT, *o*,*p*-DDE, *p*,*p′*-DDE, *o*,*p*-DDD, *p*,*p′*-DDD; endosulfans: α-, β-endosulfane, endosulfane sulfate; toxaphenes: toxaphene-26, toxaphene-50, toxaphene-62; aldrins: aldrin, dieldrin; endrin heptachlors: heptachlor, *cis*-heptachlor epoxide, *trans*-heptachlor epoxide, *trans*-nonachlor; chlordanes: α-chlordane, β-chlordane, oxychlordane; chlorobenzenes: pentachlorobenzene, hexachlorobenzene; PCBs: PCB-28, PCB-52, PCB-101, PCB-118, PCB-138, PCB-153, PCB-150.

Figure 1 lists the estimated dietary intake of pesticides, PCBs, and PFCs calculated using the USDA Food Availability Data Set. The estimated daily intake of DDT and its metabolites was 263 ng/day, driven largely by American cheese consumption (62.4 ng/day). Endosulfans and PFOA followed with 114.4 ng/day and 60 ng/day, respectively. Endosulfan intake resulted primarily from salads and cooking oils (64.5 ng/day). According to the USDA’s food availability data adjusted for loss, average daily salad and cooking oil consumption amounts to 39.5 g per person daily. PFOA intake resulted principally from meat consumption. Relatively speaking, intake from aldrin-like chemicals (aldrin, dieldrin, and endrin) was considerable (47 ng/day), due largely to dairy and vegetable products. Total PCB intake was 33 ng/day.

## Discussion

Studies that measure PFCs in consumer food are limited. A 3M-sponsored study measured PFOA, PFOS, and PFOSA in individual food samples of green beans, apples, pork, milk, chicken, eggs, bread, hot dogs, catfish, and ground beef (3M 2001). Some wildlife studies exist, describing PFCs in wild-caught fish homogenates and fillets (Ye et al. 2008a, 2008b). The 3M-sponsored study was the only previous study of PFC contamination in U.S. foods, to the best of our knowledge. Most samples had levels below the LOD (0.5 ng/g for all chemicals). The highest level of PFOA (2.35 ng/g ww) was detected in an apple purchased in Decatur, Alabama, the location of a 3M PFOA production plant. The highest level of PFOS (0.85 ng/g ww) was from milk purchased in Pensacola, Florida. The U.K. Food Standards Agency published results of PFC analysis in food collected from the 2004 Total Diet Study (Food Standards Agency 2006). PFOS exceeded the LOD in potatoes, canned vegetables, eggs, sugars, and preserves, with highest levels detected in potatoes (10 ng/g ww), including fresh potatoes as well as potato chips, french fries, and hash browns. In the U.K. study, PFOA was detected only in potatoes (1 ng/g ww). Estimated average dietary intakes for PFOS and for PFOA were 100 and 70 ng/kg/day, respectively, which for an 80-kg adult would equal approximately 8 μg/day for PFOS and 5.6 μg/day for PFOA. LODs for both PFOS and PFOA in this study were high (10–20 ng/g), which likely skewed estimated dietary intakes to higher values than actually existed given that when calculating dietary intake values below the LOD were estimated as half the LOD. A German study used duplicate diet portions to estimate total perfluorinated dietary exposure (Fromme et al. 2007). They estimated median daily dietary intake of PFOS for male and female study participants 16–45 years of age as 1.4 ng/kg body weight/day, or approximately 112 ng/day for an 80-kg individual, and 2.9 ng/kg body weight/day, or approximately 232 ng/day, for PFOA. The estimated dietary intake of PFOA in both the Fromme et al. (2007) and the U.K. Total Diet study were higher than our PFOA estimate of 60 ng/day in the present study.

A study of chemical contamination of food collected from 1992 to 2004 as part of the Canadian Total Diet Study examined PFCs, including PFOS and PFOA (Tittlemier et al. 2007). Sampling continued through 2004, although PFOS was taken off the market in 2002. PFOA was detected at the highest levels in microwave popcorn (3.6 ng/g ww) and roast beef (2.6 ng/g ww), and PFOS was detected at the highest levels in beef steak (2.7 ng/g ww) and saltwater fish (2.6 ng/g ww). PFNA was detected in the beef steak sample (4.5 ng/g ww). LODs for PFCs ranged from 0.4 to 5 ng/g ww. Estimated dietary intake of PFCs ranged from 170 to 480 ng/day, which exceed the estimated levels we measured in the present study.

Another study examined the levels of PFCs in food purchased in Catalonia, Spain, during 2006 (Ericson et al. 2008). The most commonly detected PFC was PFOS, in 24 of 36 samples, with the highest levels in an uncooked bluefish composite sample (0.654 ng/g ww), which included salmon, sardines, and tuna. PFOA was found only in whole milk, at relatively low levels (0.055 and 0.058 ng/g ww). Computed average daily intake for a standard adult man (70 kg) was estimated between 62.5 and 74.2 ng/day for PFOS. Estimated daily intakes were not calculated for the other PFCs because of a lack of detection for most of them. Another recent study examined levels of various halogenated chemicals, including pesticides, dioxins, PBDEs, and PFCs, in farm-raised fish, including salmon and tilapia (van Leeuwen et al. 2009). PFCs were not usually detected; only 41 of 429 total observations exceeded the LOD, with the highest concentration (0.6 pg/g ww) of PFOS in shrimp. Neither PFOA nor PFOS was detected in salmon or tilapia samples.

The pattern of detection of PFCs varied significantly in our study compared with previous food studies. In previous studies, typically the most commonly detected PFC was PFOS. Here, PFOS did not exceed the LOD, from 0.01 to 0.5 ng/g ww, in any samples, which is perhaps not surprising because it has been off the market since 2002. Instead, PFOA was found to exceed the LOD in 17 of 31 samples, with highest levels in butter (1.07 ng/g ww) and olive oil (1.8 ng/g ww). The relatively high levels of PFOA detected in this study might be attributed to the materials used in the processing and packaging of the food. Some food packaging materials contain trace amounts of PFOA, and PFCs have been shown to migrate from packaging materials into food oils (Begley et al. 2005). However, more research needs to be conducted to determine routes of PFC contamination of food.

A Swedish market basket survey of food samples collected in 1999 measured levels of various organohalogen contaminants, including 23 separate PCB congeners (Darnerud et al. 2006). Food homogenates were constructed based on nationwide per capita consumption data. Total dioxin-like and non-dioxin-like PCB levels were reported in composite samples for meat, fish, eggs, fats, dairy, and pastry samples. The highest levels of both dioxin-like and non-dioxin-like PCBs were detected in fish composites, with PCB-153 detected at a mean level of 2.18 ng/g ww. In the present study, we detected PCB-153 at similar levels in the salmon and canned sardine composite samples (1.21 ng/g ww and 1.83 ng/g ww, respectively) but did not detect it in any other fish samples. The estimated total lower-bound PCB dietary intake in the Darnerud et al. (2006) study was approximately 508 ng/day, which is considerably higher than our intake estimation in the present study (33 ng/day). This discrepancy could be attributed to Darnerud et al. measuring more PCB congeners than we did in this study. Additionally, 57% of the PCB intake in the Swedish study was due to fish consumption, whereas most PCB intake in the present study was due to meat consumption, reflecting the differences in diet in the two countries.

For each individual pesticide measured in this study, none of the estimated dietary intakes exceeded its corresponding U.S. EPA (2009) reference doses (RfDs), nor did the contamination levels surpass European Union maximum residue levels for pesticide residues in food (European Commission 2009). Although official RfDs have not been established for PFOA or PFOS, provisional RfDs based on rat carcinogenesis and multigenerational studies have been suggested (PFOS, 0.025 μg/kg/day; PFOA, 0.333 μg/kg/day) (Gulkowska et al. 2006). The estimated dietary intakes for either of these PFCs in this study did not approach the provisional RfDs. It is, however, worth considering possible increased effects that may result from ingesting mixtures of these chemicals. RfDs are primarily calculated through animal studies with one chemical tested at a time. By investigating single compounds, it is possible to tease apart the distinct effects due solely to that compound. However, in real life it is very rare for an individual to be exposed to only one chemical at a time. Every food within this study contained multiple pesticides. Data from the National Health and Nutrition Examination Survey serum and urine analyses support this conclusion (Bradman et al. 2007). Toxicity of mixtures of pesticides is frequently uncharacterized; toxic interaction between pesticides occurs most frequently when pesticides share cellular targets and/or metabolic pathways (Yanez et al. 2002). In these cases, interaction can occur independently as an additive process, through agonism or antagonism, or through synergy, where the combined effect is greater than the sum of the individuals (Stewart and Carter 2009). This idea of interaction is important to consider because of the high prevalence of pesticides with the same target and mechanism of toxicity.

To allow for comparisons between studies, we believe that future publications should present the raw data, including levels of detection for samples below the LOD, indicating which truly did have undetected chemical levels. We also suggest that future food and dietary intake studies that assess chemical contamination should use the current year’s USDA Food Availability Data Set instead of the 1994–1996 USDA Continuing Survey of Food Intake for Individuals. The USDA food availability data are updated yearly and are a more accurate representation of food consumption in the United States. However, the USDA Food Availability Data Set does not provide per capita intakes for every specific food type. For example, contaminant levels measured in bacon and sausage were averaged to estimate the total concentration in pork. The estimated levels might not reflect the actual levels consumed, but this is a weakness that exists in all dietary intake estimations.

USDA’s Pesticide Data Program began in 1991 to investigate pesticide residue levels in food, especially in products most frequently consumed by infants and children (USDA 2008). Overall, they found very few samples that exceeded allowable levels. Food types tested were not consistent from year to year, which prohibited annual comparisons. In 2007, the samples tested were limited to produce. None of these items was the same as foods we tested in the present study, although most pesticides tested in this study were examined by the USDA. Previous years’ programs did examine some foods common to this study; in 2006, peanut butter and poultry were tested for pesticides (USDA 2007). The Pesticide Data Program has shown that pesticide residues do exist in many food products, although in many cases residue levels were lower than those we found in this study. Our study shows that U.S. food is contaminated with a wide range of chemicals, including pesticides, PFCs, and PCBs, and that expanding the current monitoring beyond pesticides to include emerging pollutants is warranted. Further research is needed to adequately determine average levels and variability of these and other toxic chemicals in the U.S. food. Research is also needed to determine the toxicologic effects of these and other mixtures that exist in food.

## Figures and Tables

**Table 1:** Levels of marker PCBs, PFCs, and organochlorine pesticides in composite meat samples [ng/g ww or (LOD)].

Marker	Hamburger	Bacon	Sliced turkey	Sausages	Ham	Sliced chicken breast	Roast beef	Canned chili
Lipid percent	21.70	36.10	2.00	23.90	4.30	4.70	4.60	9.10
PCB-52	ND (0.1)	ND (0.09)	ND (0.04)	ND (0.1)	ND (0.05)	ND (0.05)	ND (0.04)	ND (0.03)
PCB-101	ND (0.4)	ND (0.3)	ND (0.1)	ND (0.4)	ND (0.2)	ND (0.2)	ND (0.1)	ND (0.09)
PCB-118	ND (0.2)	ND (0.1)	ND (0.06)	ND (0.2)	ND (0.07)	ND (0.08)	ND (0.09)	ND (0.05)
PCB-153	1.2	ND (0.4)	ND (0.2)	ND (0.5)	ND (0.2)	ND (0.3)	ND (0.2)	ND (0.2)
PCB-138	ND (0.7)	ND (0.4)	ND (0.2)	ND (0.6)	ND (0.2)	ND (0.3)	ND (0.2)	ND (0.2)
PCB-180	0.21	ND (0.10)	ND (0.04)	ND (0.1)	ND (0.04)	ND (0.06)	ND (0.05)	ND (0.04)
PFOA	0.15	0.24	ND (0.02)	0.09	0.02	0.02	ND (0.02)	0.02
PFBS	ND (0.04)	ND (0.05)	ND (0.02)	ND (0.04)	ND (0.02)	ND (0.02)	ND (0.02)	ND (0.01)
PFHxS	ND (0.04)	ND (0.05)	ND (0.02)	ND (0.04)	ND (0.02)	ND (0.02)	ND (0.02)	ND (0.01)
α-HCH	ND (0.08)	ND (0.03)	ND (0.01)	ND (0.03)	ND (0.01)	ND (0.01)	ND (0.01)	ND (0.01)
β-HCH	ND (0.09)	ND (0.03)	ND (0.01)	ND (0.03)	ND (0.02)	ND (0.01)	ND (0.02)	ND (0.02)
*p*,*p′*-DDT	0.08	ND (0.06)	ND (0.01)	0.17	ND (0.02)	ND (0.01)	ND (0.01)	ND (0.01)
*p*,*p′*-DDE	1.12	0.16	ND (0.03)	0.42	ND (0.04)	ND (0.02)	0.28	0.51
*o*,*p*-DDD	ND (0.03)	ND (0.03)	ND (0.01)	ND (0.03)	ND (0.01)	ND (0.01)	ND (0.01)	ND (0.01)
*p*,*p′*-DDD	0.053	ND (0.06)	ND (0.01)	0.037	ND (0.02)	ND (0.01)	ND (0.03)	ND (0.03)
Dieldrin	0.17	0.12	ND (0.02)	0.031	ND (0.02)	ND (0.01)	ND (0.02)	0.04
Endosulfan sulfate	0.17	ND (0.08)	ND (0.03)	ND (0.07)	ND (0.03)	ND (0.03)	ND (0.03)	0.10
Toxaphene-26	ND (0.2)	ND (0.08)	ND (0.04)	ND (0.08)	ND (0.04)	ND (0.04)	ND (0.04)	ND (0.03)
Toxaphene-50	ND (0.2)	ND (0.1)	ND (0.07)	ND (0.1)	ND (0.06)	ND (0.07)	ND (0.07)	ND (0.05)
α-Chlordane	ND (0.04)	ND (0.03)	ND (0.01)	ND (0.03)	ND (0.01)	ND (0.01)	ND (0.01)	ND (0.01)
Oxychlordane	ND (0.2)	ND (0.04)	ND (0.03)	ND (0.04)	ND (0.03)	ND (0.02)	ND (0.04)	ND (0.03)
*trans*-Nonachlor	0.05	ND (0.03)	ND (0.01)	ND (0.03)	ND (0.01)	ND (0.01)	ND (0.01)	ND (0.01)
*cis*-Heptachlor epoxide	0.07	ND (0.03)	ND (0.01)	0.052	ND (0.01)	ND (0.01)	ND (0.03)	ND (0.03)
Pentachlorobenzene	0.06	ND (0.03)	ND (0.02)	ND (0.03)	ND (0.02)	ND (0.01)	ND (0.02)	ND (0.01)
Hexachlorobenzene	0.18	ND (0.1)	ND (0.04)	ND (0.1)	ND (0.05)	ND (0.03)	ND (0.08)	ND (0.06)

ND, not detected.

**Table 2:** Levels of marker PCBs, PFCs, and organochlorine pesticides in composite fish samples [ng/g ww or (LOD)].

Marker	Salmon	Canned tuna	Fresh catfish fillet	Tilapia	Cod	Canned sardines	Frozen fish sticks
Lipid percent	11.90	14.80	11.60	1.60	0.30	10.30	10.30
PCB-52	0.28	ND (0.06)	ND (0.1)	ND (0.09)	ND (0.07)	0.28	ND (0.06)
PCB-101	0.51	ND (0.2)	ND (0.3)	ND (0.3)	ND (0.2)	0.67	ND (0.2)
PCB-118	0.43	ND (0.07)	ND (0.2)	ND (0.1)	ND (0.1)	0.80	ND (0.09)
PCB-153	1.21	ND (0.3)	ND (0.5)	ND (0.4)	ND (0.3)	1.83	ND (0.3)
PCB-138	0.93	ND (0.3)	ND (0.5)	ND (0.3)	ND (0.2)	1.80	ND (0.3)
PCB-180	0.44	ND (0.06)	ND (0.1)	ND (0.07)	ND (0.06)	0.49	ND (0.07)
PFOA	0.23	ND (0.05)	0.30	0.10	0.10	0.19	0.21
PFBS	ND (0.07)	ND (0.05)	ND (0.06)	ND (0.04)	0.12	ND (0.06)	ND (0.09)
PFHxS	ND (0.07)	ND (0.05)	ND (0.06)	ND (0.04)	0.07	ND (0.06)	ND (0.09)
α-HCH	0.09	ND (0.01)	0.04	0.11	0.05	0.19	0.03
β-HCH	0.06	ND (0.01)	ND (0.03)	0.11	0.05	0.12	ND (0.01)
*p*,*p′*-DDT	0.45	ND (0.01)	0.22	ND (0.05)	ND (0.02)	0.14	ND (0.03)
*p*,*p′*-DDE	3.51	0.21	9.01	0.21	0.31	2.82	0.06
*o*,*p*-DDD	0.23	ND (0.02)	0.42	ND (0.04)	0.01	0.12	ND (0.01)
*p*,*p′*-DDD	1.92	ND (0.03)	9.29	0.34	0.09	1.75	ND (0.03)
Dieldrin	1.20	ND (0.02)	2.30	ND (0.03)	0.06	0.92	ND (0.02)
Endosulfan sulfate	1.85	ND (0.04)	ND (0.1)	0.37	ND (0.08)	0.58	ND (0.03)
Toxaphene-26	0.44	ND (0.04)	0.10	ND (0.07)	0.10	0.31	ND (0.04)
Toxaphene-50	0.92	ND (0.06)	0.17	ND (0.1)	0.10	0.55	ND (0.06)
α-Chlordane	0.44	ND (0.01)	0.48	ND (0.02)	0.11	0.37	ND (0.03)
Oxychlordane	0.07	ND (0.02)	0.06	ND (0.06)	0.05	0.10	ND (0.02)
*trans*-Nonachlor	0.56	ND (0.01)	0.88	ND (0.02)	0.19	0.65	ND (0.03)
*cis*-Heptachlor epoxide	0.22	ND (0.01)	0.09	ND (0.03)	0.02	0.28	ND (0.02)
Pentachlorobenzene	0.04	ND (0.02)	ND (0.05)	ND (0.09)	ND (0.03)	0.08	ND (0.09)
Hexachlorobenzene	0.64	ND (0.05)	ND (0.2)	ND (0.2)	0.57	0.82	ND (0.1)

ND, not detected.

**Table 3:** Levels of marker PCBs, PFCs, and organochlorine in composite dairy and egg samples [ng/g ww or (LOD)].

Marker	Butter	American cheese	Other cheese	Whole milk	Ice cream	Frozen yogurt	Whole milk yogurt	Cream cheese	Eggs
Lipid percent	91.4	25.3	30.1	3.8	16.2	3.1	2.9	34	10
PCB-52	ND (0.2)	ND (0.1)	ND (0.09)	ND (0.05)	ND (0.2)	ND (0.03)	ND (0.03)	ND (0.08)	ND (0.05)
PCB-101	ND (0.2)	ND (0.3)	ND (0.3)	ND (0.2)	ND (0.5)	ND (0.09)	ND (0.08)	ND (0.2)	ND (0.1)
PCB-118	ND (0.1)	ND (0.2)	ND (0.2)	ND (0.07)	ND (0.2)	ND (0.05)	ND (0.04)	ND (0.2)	ND (0.08)
PCB-153	ND (0.5)	ND (0.4)	ND (0.4)	ND (0.2)	ND (0.5)	ND (0.1)	ND (0.1)	ND (0.4)	ND (0.2)
PCB-138	ND (0.5)	ND (0.4)	ND (0.4)	ND (0.2)	ND (0.7)	ND (0.1)	ND (0.1)	ND (0.4)	ND (0.2)
PCB-180	ND (0.1)	ND (0.1)	ND (0.10)	ND (0.05)	0.091	ND (0.03)	ND (0.03)	ND (0.09)	ND (0.05)
PFOA	1.07	ND (0.04)	ND (0.04)	ND (0.02)	ND (0.03)	ND (0.02)	ND (0.03)	ND (0.02)	ND (0.04
PFBS	ND (0.09)	ND (0.04)	ND (0.04)	ND (0.02)	ND (0.03)	ND (0.02)	ND (0.03)	ND (0.02)	ND (0.04)
PFHxS	ND (0.09)	ND (0.04)	ND (0.04)	ND (0.02)	ND (0.03)	ND (0.02)	ND (0.03)	ND (0.02)	ND (0.04)
α-HCH	ND (0.2)	ND (0.04)	ND (0.04)	ND (0.02)	ND (0.02)	ND (0.01)	ND (0.01)	ND (0.04)	ND (0.03)
β-HCH	ND (0.3)	ND (0.04)	ND (0.04)	ND (0.02)	ND (0.02)	ND (0.01)	ND (0.01)	ND (0.04)	ND (0.03)
*p*,*p′*-DDT	ND (0.1)	ND (0.04)	ND (0.04)	ND (0.02)	0.038	ND (0.02)	ND (0.01)	ND (0.04)	ND (0.03)
*p*,*p′*-DDE	5.07	4.80	0.84	0.24	1.23	0.82	0.04	5.65	0.11
*o*,*p*-DDD	ND (0.1)	ND (0.04)	ND (0.04)	ND (0.02)	ND (0.02)	ND (0.01)	ND (0.01)	ND (0.04)	ND (0.01)
*p*,*p′*-DDD	ND (0.1)	ND (0.04)	0.070	ND (0.02)	0.02	ND (0.01)	ND (0.01)	0.06	ND (0.01)
Dieldrin	1.04	0.43	0.50	ND (0.02)	0.13	0.08	0.03	0.33	ND (0.03)
Endosulfan sulfate	0.86	ND (0.2)	ND (0.1)	ND (0.05)	ND (0.2)	ND (0.03)	ND (0.03)	0.43	ND (0.03)
Toxaphene-26	ND (0.6)	ND (0.1)	ND (0.1)	ND (0.06)	ND (0.06)	ND (0.04)	ND (0.04)	ND (0.1)	ND (0.05)
Toxaphene-50	ND (0.5)	ND (0.2)	ND (0.2)	ND (0.1)	ND (0.1)	ND (0.07)	ND (0.07)	ND (0.2)	ND (0.07)
α-Chlordane	ND (0.1)	ND (0.04)	ND (0.04)	ND (0.02)	ND (0.02)	ND (0.01)	ND (0.01)	ND (0.04)	ND (0.01)
Oxychlordane	ND (0.3)	0.14	0.16	ND (0.04)	0.029	ND (0.02)	ND (0.02)	0.11	ND (0.04)
*trans*-Nonachlor	0.15	0.12	0.10	ND (0.02)	0.035	ND (0.01)	ND (0.01)	0.08	ND (0.01)
*cis*-Heptachlor epoxide	0.28	0.23	0.27	ND (0.02)	0.069	ND (0.02)	ND (0.02)	0.16	ND (0.02)
Pentachlorobenzene	0.13	ND (0.04)	ND (0.04)	ND (0.02)	ND (0.04)	ND (0.01)	ND (0.02)	ND (0.04)	ND (0.03)
Hexachlorobenzene	0.42	ND (0.2)	ND (0.2)	ND (0.07)	ND (0.1)	ND (0.05)	ND (0.05)	ND (0.2)	ND (0.07)

ND, not detected.

**Table 4:** Levels of marker PCBs, PFCs, and organochlorine in composite vegetable-based samples [ng/g ww or (LOD)].

Marker	Olive oil	Canola oil	Margarine	Cereals	Apples	Potatoes	Peanut butter
Lipid percent	100	100	79.4	0	0	0	50.5
PCB-52	ND (0.2)	ND (0.2)	ND (0.2)	ND (0.2)	ND (0.09)	ND (0.06)	ND (0.7)
PCB-101	ND (0.2)	ND (0.3)	ND (0.2)	ND (0.2)	ND (0.1)	ND (0.2)	ND (2)
PCB-118	ND (0.1)	ND (0.1)	ND (0.1)	ND (0.07)	ND (0.06)	ND (0.07)	ND (0.7)
PCB-153	ND (0.5)	ND (0.5)	ND (0.5)	ND (0.2)	ND (0.2)	ND (0.3)	ND (2)
PCB-138	ND (0.5)	ND (0.5)	ND (0.5)	ND (0.2)	ND (0.2)	ND (0.3)	ND (2)
PCB-180	ND (0.1)	ND (0.1)	ND (0.1)	ND (0.04)	ND (0.04)	ND (0.06)	0.43
PFOA	1.80	ND (0.5)	0.19	ND (0.04)	ND (0.02)	0.07	0.10
PFBS	ND (0.3)	ND (0.5)	ND (0.03)	ND (0.04)	ND (0.02)	ND (0.04)	ND (0.03)
PFHxS	ND (0.3)	ND (0.5)	ND (0.03)	ND (0.04)	ND (0.02)	ND (0.04)	ND (0.03)
α-HCH	ND (0.1)	ND (0.2)	ND (0.3)	ND (0.01)	ND (0.01)	ND (0.02)	0.20
β-HCH	ND (0.2)	ND (0.3)	ND (0.3)	ND (0.01)	ND (0.01)	ND (0.01)	0.42
*p*,*p′*-DDT	0.14	ND (0.1)	ND (0.2)	ND (0.01)	ND (0.02)	ND (0.01)	0.14
*p*,*p′*-DDE	0.49	ND (0.1)	ND (0.1)	ND (0.02)	ND (0.03)	ND (0.02)	1.25
*o*,*p*-DDD	ND (0.1)	ND (0.1)	ND (0.1)	ND (0.01)	ND (0.01)	ND (0.01)	0.08
*p*,*p′*-DDD	ND (0.1)	ND (0.1)	ND (0.1)	ND (0.01)	ND (0.01)	ND (0.01)	0.43
Dieldrin	ND (0.1)	ND (0.2)	0.24	ND (0.01)	ND (0.01)	ND (0.04)	0.55
Endosulfan sulfate	3.3	ND (0.5)	ND (0.4)	ND (0.03)	1.3	ND (0.06)	1.44
Toxaphene-26	ND (0.4)	ND (0.6)	ND (0.7)	ND (0.04)	ND (0.03)	ND (0.04)	0.39
Toxaphene-50	ND (0.5)	ND (0.5)	ND (0.5)	ND (0.07)	ND (0.06)	ND (0.07)	0.98
α-Chlordane	ND (0.1)	ND (0.1)	ND (0.1)	ND (0.01)	ND (0.01)	ND (0.01)	0.20
Oxychlordane	ND (0.2)	ND (0.3)	ND (0.3)	ND (0.02)	ND (0.03)	ND (0.03)	ND (0.2)
*trans*-Nonachlor	ND (0.1)	ND (0.1)	ND (0.1)	ND (0.01)	ND (0.01)	ND (0.01)	0.23
*cis*-Heptachlor epoxide	ND (0.1)	ND (0.1)	ND (0.1)	ND (0.01)	ND (0.01)	ND (0.02)	0.38
Pentachlorobenzene	ND (0.1)	ND (0.1)	ND (0.1)	ND (0.02)	ND (0.03)	ND (0.05)	1.22
Hexachlorobenzene	ND (0.1)	ND (0.1)	ND (0.1)	ND (0.04)	ND (0.07)	ND (0.09)	0.73

ND, not detected.
